# Disease Phenotype and Outcome Depending on the Age at Disease Onset in Patients Carrying the R92Q Low-Penetrance Variant in *TNFRSF1A* Gene

**DOI:** 10.3389/fimmu.2017.00299

**Published:** 2017-03-27

**Authors:** Estíbaliz Ruiz-Ortiz, Estíbaliz Iglesias, Alessandra Soriano, Segundo Buján-Rivas, Marta Español-Rego, Raul Castellanos-Moreira, Adrià Tomé, Jordi Yagüe, Jordi Antón, José Hernández-Rodríguez

**Affiliations:** ^1^Department of Immunology-CDB, Hospital Clinic, IDIBAPS, Barcelona, Spain; ^2^Pediatric Rheumatology Unit, Department of Pediatrics, Hospital Sant Joan de Déu, University of Barcelona, Barcelona, Spain; ^3^Rheumatology Unit, Department of Internal Medicine, Arcispedale Santa Maria Nuova – IRCCS, Reggio Emilia, Rome, Italy; ^4^Campus Bio-Medico University, Rome, Italy; ^5^Autoimmune and Systemic Diseases Unit, Department of Internal Medicine, Hospital Vall d’Hebron, Barcelona, Spain; ^6^Clinical Unit of Autoinflammatory Diseases and Vasculitis Research Unit, Department of Autoimmune Diseases, Hospital Clinic, IDIBAPS, University of Barcelona, Barcelona, Spain

**Keywords:** tumor necrosis factor receptor-associated periodic syndrome, R92Q, low-penetrance variants, autoinflammatory diseases, pediatric onset, adult onset

## Abstract

**Background:**

Tumor necrosis factor receptor-associated periodic syndrome (TRAPS) is an autosomal-dominant autoinflammatory disease caused by mutations in the *TNFRSF1A* gene. R92Q, a low-penetrance variant, is usually associated with a milder TRAPS phenotype than structural or pathogenic mutations. No studies differentiating R92Q-related disease in patients with pediatric and adult onset have been performed to date.

**Objective:**

To analyze clinical features and disease outcomes in patients diagnosed with TRAPS associated with R92Q variant and to investigate differences between patients with pediatric and adult disease onset.

**Methods:**

A retrospective review of patients with R92Q-related disease from four reference centers for autoinflammatory diseases was performed. Clinical and laboratory features, family history of autoinflammatory diseases, treatments received, and outcomes during follow-up were recorded and separately analyzed in pediatric and adult patients. Our results were included in the analysis with other reported pediatric and adult R92Q-related disease series.

**Results:**

Our series encompassed 18 patients (9 females and 9 males) with R92Q variant. In 61% of patients, disease onset occurred during infancy and in 39%, during adulthood, with a median diagnostic delay of 5 years and a follow-up of 5.4 years. A positive family history of autoinflammatory disease was detected in 28% of patients. All patients presented with febrile recurrent episodes. Other common symptoms included arthralgia/arthritis (61%), myalgia (39%), asthenia/fatigue (44%), abdominal pain (39%), headache (33%), odynophagia (33%), skin rash (28%), and chest pain (22%). During attacks, 80% of patients increased acute phase reactants levels. No patient had developed amyloidosis during the study period. At the end of follow-up, 28% of patients were asymptomatic and treatment free, 50% were receiving non-steroidal anti-inflammatory drugs or glucocorticoids on demand, and 22% were being treated with biologic agents. When differences between pediatric and adult patients were globally analyzed, adults tended to have longer attacks duration and presented more frequently with chest pain and headache, while abdominal pain, vomiting, cervical adenitis, and pharyngitis predominated in pediatric patients. No differences in outcomes and treatment requirements were observed in both age groups.

**Conclusion:**

This study has contributed to characterize R92Q-related disease by identifying trends in disease phenotypes depending on the age at disease onset.

## Introduction

The term autoinflammatory diseases was first coined in 1999 by Kastner et al. to encompass a group of clinical syndromes characterized by an increased systemic inflammatory reaction, mediated predominantly by cells and molecules of the innate immune system, and caused by mutations in genes involved in the control of inflammatory pathways (1). Among autoinflammatory diseases, tumor necrosis factor receptor-associated periodic syndrome (TRAPS; OMIM 142680), described as familial Hibernian fever in 1982 (2), was defined as an autosomal-dominant disease caused by mutations in the *TNFRSF1A* gene (located on chromosome 12p13) in 1999 (1).

Although classically TRAPS affects mostly children below 10 years of age, it can also occur in adult patients (3–9). No sex dominance has been reported (6–11). Clinical features of TRAPS include recurrent fever episodes associated with musculoskeletal symptoms, migratory rash, and ocular manifestations (5–14). Raised acute phase reactants during attacks are also typical (5, 8, 10, 11). About 10–15% of patients with TRAPS may develop amyloidosis (6–8, 10, 11, 15). Psychological stress, physical exercise, infections, menstruation, or vaccinations have been occasionally identified as trigger factors of the attacks (5).

Most of the sequence variants identified in TRAPS patients are located in the exons 2–4 (see Infevers database in http://fmf.igh.cnrs.fr/ISSAID/infevers) (16). Those missense substitutions disrupting structurally important cysteine–cysteine disulfide bonds in the extracellular domain and other mutations, such as T50M, are known as structural or pathogenic variants (5, 14). Moreover, two frequent variants, R92Q (the common name for p.Arg121Gln, located in exon 4) and P46L (also known as p.Pro75Leu, located in exon 3), are known by causing a variable TRAPS phenotype in some patients. In addition, these mutations can be observed in asymptomatic first-degree relatives and in healthy individuals (8, 14). For these reasons, R92Q and P46L have been recently classified as variants of uncertain significance (17). However, the potential pathogenic role of low-penetrance mutations causing TRAPS or TRAPS-like phenotypes still generates controversy among investigators (5, 6, 9, 12, 13).

Several studies have reported that patients carrying R92Q tend to present with milder disease phenotype and better long-term prognosis compared with those carrying structural or pathogenic *TNFRSF1A* mutations, who usually suffer from more severe manifestations and long-term complications (e.g., amyloidosis) (4–11, 18). While structural mutations are typically observed in children, those patients with adult onset of TRAPS more often carry the R92Q variant (4, 8).

Studies focused on R92Q-related disease are scarce. In addition, no studies on TRAPS associated with R92Q variant (or R92Q-related disease) differentiating disease phenotype, treatment requirements, and outcomes according to the age at disease onset (pediatric and adult) have been performed to date. Therefore, we aimed to investigate clinical and laboratory features, therapeutic approaches, and long-term outcomes in a cohort of pediatric and adult patients carrying the R92Q low-penetrance variant in *TNFRSF1A* gene, with special interest in the analysis of differences between the two age groups. Previous pediatric and adult case series of patients with R92Q-related disease reported in the literature were also reviewed and used for final comparisons.

## Materials and Methods

### Patients’ Selection and Data Collection

From January 2006 to June 2016, we retrospectively reviewed medical charts of pediatric and adult patients diagnosed with an autoinflammatory disease attributed to R92Q variant in *TNFRSF1A* gene, in four reference centers for autoinflammatory diseases (Clinical Unit of Autoinflammatory Diseases, Departments of Autoimmune Diseases and Immunology, Hospital Clínic of Barcelona, Barcelona, Spain; Pediatric Rheumatology Unit, Department of Pediatrics, Hospital Sant Joan de Déu, Barcelona, Spain; Rheumatology Unit, Department of Internal Medicine, Arcispedale Santa Maria Nuova – IRCCS, Reggio Emilia, Italy; and Autoimmune and Systemic Diseases Unit, Department of Internal Medicine, Hospital Vall d’Hebron, Barcelona, Spain).

Patients were included if an autoinflammatory disease was suspected after ruling out autoimmune, infectious, or malignant causes, and R92Q low-penetrance variant in *TNFRSF1A* gene was found. Patients with structural variants in *TNFRSF1A* gene or with concomitant mutations in *MEFV, MVK*, and *NLRP3* genes were excluded to avoid potential confounding factors for a more accurate diagnosis.

The recently published provisional Eurofever classification criteria for autoinflammatory diseases (9) were used to assess the level of agreement with TRAPS diagnosis in our patients. Variables (and their values) for TRAPS classification included: duration of episodes more than 6 days (19 points), presence of periorbital edema (21 points) or migratory rash (18 points), and also absence of vomiting (14 points) or oral aphthae (15 points). In patients with structural mutations, a cut-off value ≥43 was reported to yield 80% sensitivity and 91% specificity for TRAPS classification (9). Of note, in the same study, these criteria were associated with lower sensitivity and specificity (59 and 84%, respectively) for patients carrying the R92Q variant, although 52% of them could be still classified as TRAPS (9).

Clinical features recorded included frequency and duration of attacks, and presence of fever, arthralgia/arthritis, myalgia, abdominal and chest pain, cutaneous rash, ocular symptoms, and other less frequent manifestations (Table 1). Type of medications used and response to them, at disease onset and during follow-up, as well as their continuous or on-demand administration, were also collected. Laboratory parameters included complete blood cell counts, C-reactive protein (CRP) and/or serum amyloid A (SAA) levels, erythrocyte sedimentation rate (ESR), urinalysis with proteinuria, and markers of autoimmunity, such as antinuclear antibodies, rheumatoid factor, and complement levels. Genetic testing of the most common genes causing monogenic autoinflammatory diseases (*TNFRSF1A* for TRAPS, *MEFV* for familial Mediterranean fever, *MVK* for mevalonate kinase deficiency, and *NLRP3* for cryopyrin-associated periodic syndromes) was carried out.

**Table 1:** **Demographic, clinical, and laboratory features of patients with R92Q variant in our study series**.

	A	B	C	*p* Value (A vs. B)

	R92Q patients with pediatric onset (*n* = 11)	R92Q patients with adult onset (*n* = 7)	All R92Q patients (*n* = 18)	
**Demographic data**				
Sex (female/male)	4/7	5/2	9/9	0.34
Age at symptoms onset (years)	7.6; 8 (1–15)	25; 23 (16–43)	14.3; 12 (1–43)	0.004
Age at diagnosis (years)	12; 12 (5–16)	31; 25 (16–48)	19; 16 (5–48)	0.015
Time from disease onset to diagnosis (years)	4.1; 4 (0.3–9)	5.8; 2 (0.3–25)	5; 3 (0.3–25)	0.65
Follow-up (years)	6.2; 6 (2–10)	4; 5 (1–8)	5.4; 5.5 (1–10)	0.18
Positive family history	4 (36)	1 (14)	5 (28)	0.60
TRAPS Eurofever classification criteria^a^	4 (36)	6 (86)	10 (56)	0.066
**Clinical features^b^**				
Fever (≥38°C)	11 (100)	7 (100)	17 (100)	1
Asthenia/fatigue	6 (55)	2 (29)	8 (44)	0.37
Arthralgia/arthritis	6 (55)	5 (71)	11 (61)	0.64
Myalgia	4 (36)	3 (43)	7 (39)	1
Abdominal pain	5 (46)	2 (29)	7 (39)	0.63
Vomiting	1 (9)	0 (0)	1 (6)	1
Chest (pleuro-pericardial) pain	1 (9)	3 (43)	4 (22)	0.25
Skin rash	2 (18)	3 (43)	5 (28)	0.33
Headache	3 (27)	3 (43)	6 (33)	0.63
Conjunctivitis	2 (18)	1 (14)	3 (17)	1
Periorbital edema	1 (9)	1 (14)	2 (11)	1
Cervical adenitis	3 (27)	0 (0)	3 (17)	0.25
Pharyngitis/odynophagia	4 (36)	2 (29)	6 (33)	1
Oral aphthae	2 (18)	1 (14)	3 (17)	1
**Attacks characteristics^c^**				
Duration (days)	22; 4 (2–160)	35; 21 (4–90)	27; 11 (2–160)	0.056
Frequency (per year)	12; 6 (1.5–50)	5; 6 (0.3–8)	9; 6 (0.3–50)	0.20
**Laboratory (during attacks)^d^**				
CRP >1.5 mg/dL and/or SAA >6.4 mg/dL	6/8 (75)	6/7 (86)	12/15 (80)	1
ESR >20 mm first hour	4/8 (50)	2/4 (50)	6/12 (50)	1
Leukocyte count >11,000/mm^3^	3/8 (38)	2/5 (40)	5/13 (38)	1
Hemoglobin <120 mg/L	2/8 (25)	0/5 (0)	2/13 (15)	0.5
Platelets count >350,000/mm^3^	0/7 (0)	2/5 (40)	2/12 (17)	0.15
Proteinuria (absence) at the end of follow-up	10/10 (100)	6/6 (100)	15/15 (100)	1
**Other studied genes (negative/performed)**				
*MEFV*	11/11 (100)	6/6 (100)	17/17 (100)	1
*MVK*	9/9 (100)	7/7 (100)	16/16 (100)	1
*NLRP3*	8/8 (100)	6/6 (100)	14/14 (100)	1

*CRP, C-reactive protein; ESR, erythrocyte sedimentation rate; MEFV, Mediterranean fever gene; MVK, mevalonate kinase gene; NLRP3, nod-like receptor family pyrin domain containing 3 gene; TRAPS, tumor necrosis factor receptor-associated periodic syndrome*.

*Continuous values are given as mean; median (range)*.

*^a^Patients achieving provisional Eurofever classification criteria for TRAPS (9)*.

*^b^Values as total number of patients and %*.

*^c^Because a remarkable intrasubject variability with regard to duration and frequency of attacks was found in the majority of patients, only the highest values were used for calculations*.

*^d^Abnormal values during attacks, later normalized (from available results)*.

This retrospective study was approved by the Research Ethics Committee of the Hospital Clínic of Barcelona. Patients’ information was dissociated prior to analysis, and all procedures were performed in accordance with the ethical principles expressed in the 2013 Declaration of Helsinki.

### Groups Based on Age at Disease Onset and Review of the Literature

Based on previous studies, patients aged <16 and ≥16 years at disease onset were considered to have pediatric and adult disease onset, respectively (4, 11, 13). In addition, those cohort series of patients with R92Q-related disease reported until 2016 (identified through PubMed search) with consistent data about clinical manifestations and outcomes during the follow-up, which also provided separate information with regard to the age of disease onset (pediatric and adult), were compared with our case series and used for global calculations.

### Statistical Analysis

Results (in text and tables) are expressed as mean ± SD or median plus range, where applicable. Chi-square or Fisher’s exact tests were used for contingency tables. Quantitative differences between groups were analyzed by using Student’s unpaired *t*-test. Data were analyzed with the SPSS PC statistical package (version 20.0). Differences with a value of *p* < 0.05 were considered statistically significant.

## Results

### Overall Characteristics of Patients of All Ages with R92Q-Related Disease

A total of 18 patients with R92Q variant in *TNFRSF1A* gene following inclusion criteria were analyzed. Seven patients were excluded because of carrying the R92Q variant and other concomitant mutations in *NLRP3* and *MEFV* genes, or they presented with a disease phenotype permitting a different definite diagnosis. None of the included patients met diagnostic criteria for periodic fever, aphthous stomatitis, pharyngitis, and adenitis syndrome (19).

When provisional Eurofever classification criteria for TRAPS (9) were applied in our patients, 10 (56%) of them reached the cut-off for TRAPS classification. Among these, four (36%) were children and six (86%) adult patients (*p* = 0.066).

Nine (50%) patients were female and nine (50%) were male. In 11 (61%) patients, disease onset occurred during infancy, at a mean age of 7.6 years (median 8 years; range 1–15 years); and in seven (39%) patients, symptoms started during adulthood, at a mean age of 25 years (median 23 years; range 16–43 years). Seven (39%) and 11 (61%) patients were diagnosed during pediatric and adult age, respectively. Overall, mean diagnostic delay was 5 years (median 3 years; range 4 months–25 years).

All patients presented with febrile recurrent episodes with disease-free intervals. A remarkable intersubject and intrasubject variability was observed with regard to duration and frequency of attacks (Table 1). The most common symptoms accompanying fever episodes were arthralgia/arthritis (61%), myalgia (39%), asthenia/fatigue (44%), abdominal pain (39%), headache (33%), odynophagia (33%), skin rash (29%), and chest pain (22%). Other less frequent manifestations included facial/periocular edema, oral aphthae, cervical adenitis, and conjunctivitis. A positive family history of an autoinflammatory (R92Q-related) disease was detected in five (28%) patients (four children and one adult).

During attacks, 80% of patients had increased CRP or SAA levels and 50% of them showed high ESR values. However, hemoglobin levels and leukocyte and platelet counts were abnormal in less than half of patients during attacks. At the end of follow-up, proteinuria or other AA amyloidosis signs were not observed in any of our patients. With regard to genetic results, all patients included in the study carried R92Q low-penetrance variant in *TNFRSF1A* gene (16 of them as heterozygous mutations and two in homozygosity), and no other mutations were identified in *MEFV* (17 patients), *MVK* (16 patients), or *NLRP3* (14 patients) genes.

The main demographic, clinical, and laboratory features of all patients with R92Q variant from our patients are depicted in Table 1. Data from the main (all retrospective) series with mixed pediatric and adult results published to date are shown in Table 2.

**Table 2:** **Demographic, clinical, and laboratory features of the main series combining adult and pediatric patients with R92Q-related disease**.

	Hull et al. (10)	Ravet et al. (8)	Gattorno et al. (12)^b^	Lachmann et al. (5)^c^	Federici et al. (9)^d^	Ruiz-Ortiz et al. (present series)
**Demographic data**						
*N*	9	34	15	54	78	18
Sex (female/male)	3/6	17/17	–	–	38/40	9/9
Age at symptoms onset (years)^a^	22 (<1–53)	19	58 ± 64	6 (0–53)	6 (3–19)	12 (1–43)
Age at diagnosis (years)^a^	–	–	–	–	–	16 (5–48)
Time from disease onset to diagnosis (years)^a^	–	–	–	–	6.4 (3.4–25.9)	3 (0.3–25)
Follow-up (years)^a^	–	–	–	–	13	5.5 (1–10)
Positive family history (%)	–	21	7	19	–	28
**Clinical features (%)**						
Fever (≥38°C)	100	48	100	94	100	100
Asthenia/fatigue	–	–	–	–	72	44
Arthralgia/arthritis	89	48	17	66	65	61
Myalgia	89	48	53	66	28	39
Abdominal pain	56	39	60	66	59	39
Vomiting	–	–	40	26	26	6
Chest (pleuro-pericardial) pain	33	32	13	22	24	22
Skin rash	78	36	33	30	20	28
Headache	–	16	53	39	5	33
Conjunctivitis	100	6	13	17	20	17
Periorbital edema	78	12	7	17	19	11
Cervical adenitis/lymphadenopathy	–	19	60	25	26	17
Pharyngitis/odynophagia	–	12	67	24	22	33
Oral aphthae	–	–	40	14	15	17
**Attacks characteristics**						
Duration (days)	16 (6–30)	7.4	4.7 ± 3.7	–	–	11 (2–160)
Frequency (per year)	11 (9–>12)	–	–	–	–	6 (0.3–50)
**Increased inflammatory markers during attacks (%)**	100	100	–	–	–	80
**Other studied genes (negative/performed)**	–	MEFV (some positive)	MEFV, MVK	MEFV (22/22), MVK (11/11), NLRP3 (2/2)	–	MEFV (17/17), MVK (16/16), NLRP3 (14/14)
**Amyloidosis development (%)**	0	6.2	–	0	–	0

*^a^Continuous results as mean or median, plus SD or range (when available)*.

*^b^Data from 15 patients with *TNFRSF1A* low-penetrance variants; among them, 13 (87%) patients carried R92Q (12)*.

*^c^Data from 59 patients with *TNFRSF1A* P46L and R92Q variants; among them, 54 (91.5%) patients carried R92Q (5)*.

*^d^Data from 78 patients with *TNFRSF1A* low-penetrance mutations, no mutations or genetic test not done or P46L and R92Q variants; among them, 57 (73%) patients carried R92Q (9)*.

Global results from series with patients of all ages with R92Q-related disease (5, 8–10, 12), including ours, confirmed no sex preference and a family history of an autoinflammatory (R92Q-related) disease was recorded in 7–28% of cases (5, 8, 12). The age at disease onset ranged from less than 1 year to the fourth to sixth decades. The mean/median of attacks duration varied between 4.7 and 16 days (8, 10, 12) and the frequency of attacks between 6 and 11 episodes per year (10) (with marked inter- and intra-study variability for both parameters). Fever was present in almost 100% of patients (5, 9, 10, 12), except in one study that showed lower prevalence (8). Arthralgia/arthritis was present in about half to two-thirds of patients, myalgia in 39–66%, abdominal pain in 39–66% (5, 8–10, 12), vomiting in 6–40% (5, 9, 12), chest/pleuro-pericardial pain in 22–33%, skin rash in 20–36%, headache in 16–53%, conjunctivitis in 6–20%, periorbital edema in 7–19%, cervical adenitis or lymphadenopathy in 17–26%, odynophagia or pharyngitis in 12–33%, and oral aphthosis in 14–40% of cases (5, 8–10, 12). Of note, the study by Hull et al. (10) reported a higher frequency of joint, muscular, skin, and ocular involvement than the other studies (Table 2). Between 80 and 100% of patients had elevated acute phase reactants (mainly CRP and/or SAA) during attacks (8, 10). Overall, results from our study group did not differ with those from the previously reported series.

### Differences between Patients with Pediatric and Adult Onset of R92Q-Related Disease

No statistically significant differences with regard to clinical and laboratory features at disease presentation were found in the present series between pediatric and adult patients (Table 1). However, when previous studies on R92Q-related disease including patients with pediatric (7, 11, 13) and adult onset (6, 7) were analyzed together with our results (Table 3), no sex predominance was observed and a positive family history of R92Q-related disease tended to be higher in pediatric (4–50%) than in adult patients (6–17%). The mean/median age at disease onset was of 3.6–8 years in children and 23–28.8 years in adults. Duration of attacks tended to be longer in adult patients (mean/median from 7 to 21 days) than in children (from 4 to 9 days). Frequency of attacks was similarly heterogeneous in both groups. Among clinical features, fever was equally present in almost all patients, in all studies. Arthralgia/arthritis, myalgia, skin rash, ocular symptoms, and oral aphthae occurred in a similar proportion in both groups. Chest pain was consistently reported in almost half of adult patients and less frequently in most pediatric studies. Headache was also observed in a higher proportion in adults (42–43%) than in children (20–30%). Conversely, abdominal pain was more often presented by children (40–67%) than by adults (25–33%). Vomiting was not reported in adult series but occurred in 9–30% of pediatric patients. Cervical adenitis or lymphadenopathy (27–65 vs. 0–19%) and pharyngitis or odynophagia (13–65 vs. 11–29%) were predominantly observed in children (6, 7, 11, 13).

**Table 3:** **Characteristics of patients with R92Q low-penetrance *TNFRSF1A* variants in studies including patients with pediatric and adult onset**.

	Pediatric-onset series				Adult-onset series		
	Dodé et al. (7)	Lainka et al. (13)^b^	Pelagatti et al. (11)	Ruiz-Ortiz et al. (present series)	Dodé et al. (7)	Cantarini et al. (6)^c^	Ruiz-Ortiz et al. (present series)
**Demographic data**							
Patients (*N*)	6	15	20	11	6	25	7
Sex (female/male)	3/3	–	9/11	4/7	1/5	17/19	5/2
Age at symptoms onset (years)^a^	7.3 (2–15)	5 (1–14)	3.6 (0.6–13)	8 (1–15)	28.8 (22–36)	26.6 ± 15	23 (16–43)
Age at diagnosis (years)^a^	–	7 (1–16)	6.1 (1.2–15)	12 (5–16)	–	–	25 (16–48)
Time from disease onset to diagnosis (years)^a^	–	–	–	4 (0.3–9)	–	–	2 (0.3–25)
Follow-up (years)^a^	–	–	7.3 (1.7–14.3)	6 (2–10)	–	12.7 ± 11.3	5 (1–8)
Positive family history (%)	50	–	4	36	17	6	14
**Clinical features (%)**							
Fever (≥38°C)	100	100	100	100	100	97	100
Asthenia/fatigue	–	–	–	55	–	–	29
Arthralgia/arthritis	17	53	40	55	17	55	71
Myalgia	–	27	35	36	–	55	43
Abdominal pain	67	40	40	46	33	25	29
Vomiting	–	20	30	9	–	–	0
Chest (pleuro-pericardial) pain	50	20	4	9	50	50	43
Skin rash	50	33	20	18	17	19	43
Headache	–	20	30	27	–	42	43
Conjunctivitis	–	13	10	18	–	19^d^	14
Periorbital edema	–	–	0	9	–	19^d^	14
Cervical adenitis/lymphadenopathy	–	40	65	27	–	19	0
Pharyngitis/odynophagia	–	13	65	36	–	11	29
Oral aphthae	–	–	35	18	–	25	14
**Attacks characteristics**							
Duration (days)	6 (1–20)	9 (2–24)	5.9 (3–15)	4 (2–160)	7.5 (2–20)	>7 (69%)	21 (4–90)
Frequency (per year)	20 (6–30)	–	10.3 (3–20)	6 (1.5–50)	27.6 (6–48)	7 ± 3.9	6 (0.3–8)
**Increased inflammatory markers during attacks (%)**	–	100	100	75	–	100^e^	86
**Other studied genes (negative)**	MEFV	MEFV, MVK	MEFV, MVK	MEFV, MVK, NLRP3	MEFV	MEFV, MVK, NLRP3, NLRP12	MEFV, MVK, NLRP3
**Amyloidosis development (%)**	17	–	0	0	0	0	0

*^a^Continuous results as mean or median, plus SD or range (when available)*.

*^b^Data obtained from 15 patients with R92Q variants in *TNFRSF1A* gene (13)*.

*^c^Data from 36 patients with *TNFRSF1A* low-penetrance variants; among them, 25 (69%) patients carried R92Q (6)*.

*^d^Overall value for ophthalmological abnormalities (which included the presence of conjunctivitis and/or periorbital edema) in this series was 19%*.

*^e^All patients had high serum amyloid A values but normal C-reactive protein levels*.

### Disease Outcomes and Therapeutic Approaches during Disease Course

Patients in our study were followed for a mean of 5.4 years (median 5.5 years; range 1–10 years). Only two recent studies have also reported information from patients with R92Q-related disease after a long-term follow-up period (6, 11) (Table 3). Therapeutic strategies in ours and those previous studies similarly included the use of non-steroidal anti-inflammatory drugs (NSAIDs), colchicine, oral glucocorticoids (usually at a dose equivalent to ≥0.5 mg/kg/day of prednisone), and biologic agents (anakinra and etanercept, as IL-1 and TNF blockers, respectively).

Medications used in our cohort are illustrated in Table 4. As starting treatment, NSAIDs on demand were used in 6 patients, colchicine in 2 patients, glucocorticoids in 11 patients (5 with continuous and 6 with on-demand administration), and etanercept and anakinra, in 1 and 2 patients, respectively. At the end of follow-up, five (28%) patients were asymptomatic and treatment free (representing 18% of pediatric and 43% of adult patients), five (28%; 36% of pediatric and 14% of adult patients) continued receiving on-demand NSAIDs, four (22%; 27% of pediatric and 14% of adult patients) were on glucocorticoids on demand, and four (22%; 18% of pediatric and 28% of adult patients) were treated with biologic agents (one with weekly etanercept, and two and one with anakinra, daily and on demand, respectively).

**Table 4:** **Treatment characteristics at baseline and at the end of follow-up**.

	R92Q patients with pediatric onset (*n* = 11)	R92Q patients with adult onset (*n* = 7)	All R92Q patients (*n* = 18)
**Initial treatment**			
NSAIDs	3 (OD)	3 (OD)	6 (OD)
Colchicine	1 (C)	1 (C)^c,d^	2 (C)
Glucocorticoids	5 (OD); 2 (C)^a,b^	1 (OD); 3 (C)^c,d,e^	6 (OD); 5 (C)
Biological agents	1 AN (C)^a^; 1 ET (C)^b^	1 ET/AN (C)^d^	1 AN (C); 1 ET (C); 1 ET/AN (C)
**Treatment at the end of follow-up**			
No treatment	2	3	5
NSAIDs	4 (OD)	1 (OD)	5 (OD)
Colchicine	0	0	0
Glucocorticoids	3 (OD)	1 (OD)	4 (OD)
Biological agents	1 AN (OD)^a^; 1 ET (C)^b^	1 AN (OD)^d^; 1 AN (C)^e^	3 AN (2 OD, 1 C); 1 ET (C)

*AN, anakinra; C, continuous; ET, etanercept; OD, on demand; NSAIDs, non-steroidal anti-inflammatory drugs*.

*Patients ^a^ and ^b^ initially received glucocorticoids and a biological agent; and at the end of follow-up, patient ^a^ is receiving anakinra on demand and patient ^b^, etanercept 50 mg/week. Patient ^c^ received colchicine and glucocorticoid therapy. Patient ^d^ was treated with colchicine, prednisone, etanercept, and finally, anakinra (on an initial continuous administration, which could be switched to on demand during the disease course). Patient ^e^ started with continuous glucocorticoids and was later switched to anakinra*.

Previous data on disease outcomes and treatment used at the end of follow-up in a series of pediatric patients with R92Q-related disease revealed that 25% of cases had shown spontaneous resolution of symptoms, 12 and 44% were being treated with NSAIDs and glucocorticoids on demand, respectively, and 18% with continuous glucocorticoids or biologic agents (11). In a previous adult series, at the end of the study, about 8% of cases were receiving only NSAIDs, 46% glucocorticoids on demand, 23% continuous glucocorticoids, 3% colchicine, and 19% were being treated with cytokine blockers (6).

Although these studies analyzed small number of patients, all of them showed similar trends regarding disease outcomes and therapeutic strategies utilized, without clear differences between pediatric and adult patients.

## Discussion

R92Q has been recently classified by a panel of expert clinicians and geneticists in autoinflammatory diseases as a variant of uncertain significance because its common presence in the general population and by the fact that this variant does not segregate with the phenotype in members of the same family (17). Compared to patients with *TNFRSF1A* structural or pathogenic mutations, patients carrying the R92Q variant exhibit milder disease presentation and disease outcome, shorter febrile episodes, lower intensity and frequency of typical symptoms, a considerably lower or inexistent risk for developing amyloidosis, and a later (even during adulthood) disease onset (4, 6, 8, 10, 11, 14). Despite of this milder phenotype, almost all patients with R92Q variant are usually treated with (on demand or continuous) NSAIDs or glucocorticoids, and a remarkable proportion (up to 20%) of them may also require biologic therapy (4, 6, 11, 13). In addition, R92Q variant has been found in a higher proportion of patients with autoinflammatory symptoms than in several control populations (6, 7, 13–15). For these reasons, many expert clinicians still consider R92Q a low-penetrance mutation rather than a polymorphism (4, 6–8, 10, 12–15).

Definite diagnosis of TRAPS usually relies on the presence of suggestive clinical features supported by the existence of functional mutations in the *TNFRSF1A* gene. In this regard, the recent TRAPS provisional Eurofever classification criteria for patients with structural or pathogenic mutations yielded reasonable sensitivity and specificity (9). The same validation study showed a remarkable lower sensitivity and specificity for patients carrying the R92Q variant, but at least 52% of R92Q patients reached the cut-off for TRAPS classification (9). Similarly, 56% of our patients with R92Q-related disease could be also classified as having TRAPS. Interestingly, this proportion was considerably higher for our adult patients (86%) and lower for our children (30%). The good concordance with the original TRAPS validation study for our patients carrying R92Q makes our results reliable, particularly for those patients with adult disease onset.

Clinical and laboratory features from previous series including R92Q patients of all ages (5, 8–10, 12) are equivalent to those found in our series. When patients with R92Q-related disease are compared with those carrying structural *TNFRSF1A* mutations, a family history of an autoinflammatory (R92Q-related) disease and the presence of myalgia, abdominal pain, and ocular symptoms are more frequently observed in the group with structural variants, and pharyngitis/odynophagia and oral aphthosis predominate in patients with R92Q variant (5, 9). The remaining symptoms are similarly presented by patients with structural and R92Q variants (5, 9). While a variable proportion of patients carrying structural variants may develop amyloidosis over time, those carrying R92Q are at a very low (or absent) risk for developing this complication (5).

When patients with R92Q-related disease with onset during childhood (7, 11, 13) are compared with those initiating symptoms during adulthood (6, 7), a positive family history of an autoinflammatory disease seems to predominate in pediatric patients. No sex dominance exists. Although the age at disease onset shows wide dispersion, most children usually present with disease-related symptoms at 3–7 years of age, and among adults, symptoms often start during the second decade of life. Duration of attacks seems to be longer in adults, but heterogeneity in duration and frequency of attacks is equally observed in both groups. With regard to clinical manifestations, fever is shared by almost all patients; and musculoskeletal, cutaneous, and ocular symptoms are common features similarly present in both age groups; oral aphthosis occurs in a lower proportion, with no differences between children and adults. However, pleuro-pericardial/chest pain and headache are more frequently observed in adult patients than in children. Conversely, other features, such as abdominal pain, vomiting, cervical adenitis/lymphadenopathy, and pharyngitis/odynophagia, seem to be predominant in pediatric patients (6, 7, 11, 13).

Although no treatment guidelines for autoinflammatory diseases have been elaborated yet, therapeutic approaches for all these conditions aim to control symptoms and prevent attacks and long-term complications. Previous investigations have documented differences in disease outcomes and treatments used in TRAPS patients carrying structural variants compared to those carrying R92Q (or other low-penetrance mutations) (6, 11). While patients with structural mutations usually have a chronic and relapsing course, with an increased risk for developing amyloidosis, and the majority of them also require biologic therapy (mainly IL-1 and TNF blockers) (6, 11), most patients with R92Q-related disease can be treated with NSAIDs or glucocorticoids on demand only (6, 11), and in about 25% of patients (pediatric and adults), symptoms may evolve to spontaneous resolution during the course of the disease, without requiring any treatment (11). However, continuous or on-demand administration of biologic agents can be indicated in about 20% of cases (6, 11).

The molecular mechanisms responsible for clinical phenotypes of TRAPS and the role of structural or pathogenic mutations and those low-penetrance variants, such as R92Q, still remain to be completely elucidated. While forms of *TNFRSF1A* cysteine mutations clearly destabilize the protein structure and produce defects in cell surface expression and TNF binding, R92Q mutants, which share structural similarities with the wild-type protein, also share similar mechanisms of action with wild-type *TNFRSF1A* (20). In this sense, structural mutations have also shown to produce deeper disturbances in T-cell function than R92Q and other low-penetrance variants (21). However, recent investigations have demonstrated a constitutive activity of R92Q mutants associated with TRAPS, which might be explained by configurational changes induced after ligand binding that can act as the trigger for TNFR1 signaling. Indeed, these structural changes are not present in wild-type receptors (22). Other data, in patients with multiple sclerosis, point toward the enhancement of the interaction between the receptor and its ligand by the R92Q variant, resulting in the potentiation of TNF-mediated pathways (23). In addition, several authors have postulated a potential role of low-penetrance *TNFRSF1A* variants (including R92Q, P46L, and other recently reported, such as V95M, D12E, and R104Q) in causing different autoinflammatory phenotypes (6, 21). These low-penetrance variants might also contribute, as a possible susceptibility factor in the development of multifactorial polygenic inflammatory or autoimmune conditions, such as idiopathic recurrent acute pericarditis, Behçet’s disease, juvenile idiopathic arthritis, and other autoinflammatory diseases (6, 14, 24–27).

This study has several limitations that include (a) the present study and all the previous series on TRAPS and R92Q-related disease published to date are retrospective and included a relatively small number of patients; (b) very few of them differentiated patients with pediatric or adult disease onset; (c) most TRAPS series studied patients with R92Q together with other low-penetrance variants, thus providing mixed results; (d) the small number of patients analyzed, and the heterogeneity in some variables and results, make comparisons mainly estimative between patients carrying structural and low-penetrance mutations and between R92Q carriers of different ages; and (e) pharmacological treatments used in all the studies were guided by personal experience, and since no evidence-based therapeutic protocols have been elaborated yet, no strong recommendations can be made in this regard. However, we consider that the present study also has several strengths: (a) our results were in concordance with general R92Q-related disease series including patients of all ages, and with those studies focused in patients with pediatric and adult onset; (b) our R92Q patients achieved criteria for TRAPS classification in a similar proportion than the R92Q patients included in the original Eurofever validation study (9), which was also comparable with TRAPS caused by structural mutations for our adult patients with R92Q variant.

In summary, this study has contributed to characterize TRAPS associated with R92Q variant, particularly in differentiating clinical phenotypes according to the age at disease onset. Adult patients tend to have longer duration of attacks and exhibit more frequently chest pain and headache, than pediatric patients. Abdominal pain, vomiting, cervical adenitis/lymphadenopathy, pharyngitis/odynophagia, and a family history of an autoinflammatory disease seem to predominate in pediatric patients. With independence of the age at disease onset, most patients with R92Q-related disease usually receive on demand NSAIDs and glucocorticoids, and about a quarter part of them may have a resolution of symptoms over time, without requiring treatment. However, up to 20% of patients may still need biologic agents (IL-1 or TNF blockers) at the end of follow-up to control disease activity.

The present results evidence the level of clinical and genetic complexity of TRAPS phenotype caused by the R92Q variant and also lead to emphasize the core importance of interpreting genetic results in an appropriate clinical context. In order to corroborate these findings and to achieve a better understanding of TRAPS spectrum, further studies including a large number of patients with TRAPS, caused by structural mutations, and also by R92Q and other low-penetrance *TNFRSF1A* variants, are granted. In addition, whether or not this subset of patients should undergo whole exome sequencing to search for concomitant disease-causing mutations in unknown genes may be a matter of discussion.

## Author Contributions

*Category 1*: conception and design of study: ER-O and JH-R; acquisition of data: ER-O, EI, AS, SB, ME-R, RC-M, AT, and JH-R; analysis and/or interpretation of data: ER-O, ME-R, and JH-R. *Category 2*: drafting the manuscript: ER-O and JH-R; revising the manuscript critically for important intellectual content: ER-O, EI, AS, SB, JY, JA, and JH-R. *Category 3*: approval of the version of the manuscript to be published: ER-O, EI, AS, SB, ME-R, RC-M, AT, JY, JA, and JH-R.

## Conflict of Interest Statement

The authors declare that the research was conducted in the absence of any commercial or financial relationships that could be construed as a potential conflict of interest.

## Abbreviations

TNF, tumor necrosis factor; TRAPS, tumor necrosis factor receptor-associated periodic syndrome.
